# Dual-stent thrombectomy for recanalization of cerebral embolism caused by infective endocarditis: a case report

**DOI:** 10.3389/fneur.2024.1484492

**Published:** 2024-12-10

**Authors:** Haiqi Zhang, Jianfei Chen, Wansheng Chang, Feng Lin, Jijun Yin

**Affiliations:** Department of Neurology, The Second People’s Hospital of Liaocheng, The Second Hospital of Liaocheng Affiliated to Shandong First Medical University, Linqing, China

**Keywords:** dual-stent thrombectomy, infective endocarditis, large-vessel occlusion, valve replacement surgery, clinical outcomes

## Abstract

**Methods:**

In this case report, we present an in-depth narrative of a patient who was subjected to mechanical thrombectomy (MT) for an obstruction in the main trunk and bifurcation of the left middle cerebral artery subsequent to Infective Endocarditis (IE). Initial intervention using a solitary-stent technique proved to be ineffective; thus, we shifted to a dual-stent strategy, which successfully recanalized the compromised blood vessel.

**Results:**

The dual-stent retriever method can be especially advantageous for treating persistent clots that occur at arterial bifurcations resisting the efforts of a single-stent retriever during the MT process.

**Conclusion:**

Dual-stent thrombectomy increases the likelihood of clot extraction due to its ability to encompass a larger area of the thrombus within the stent’s framework, potentially improving the clinical outcomes.

## Background

**Figure 1:**
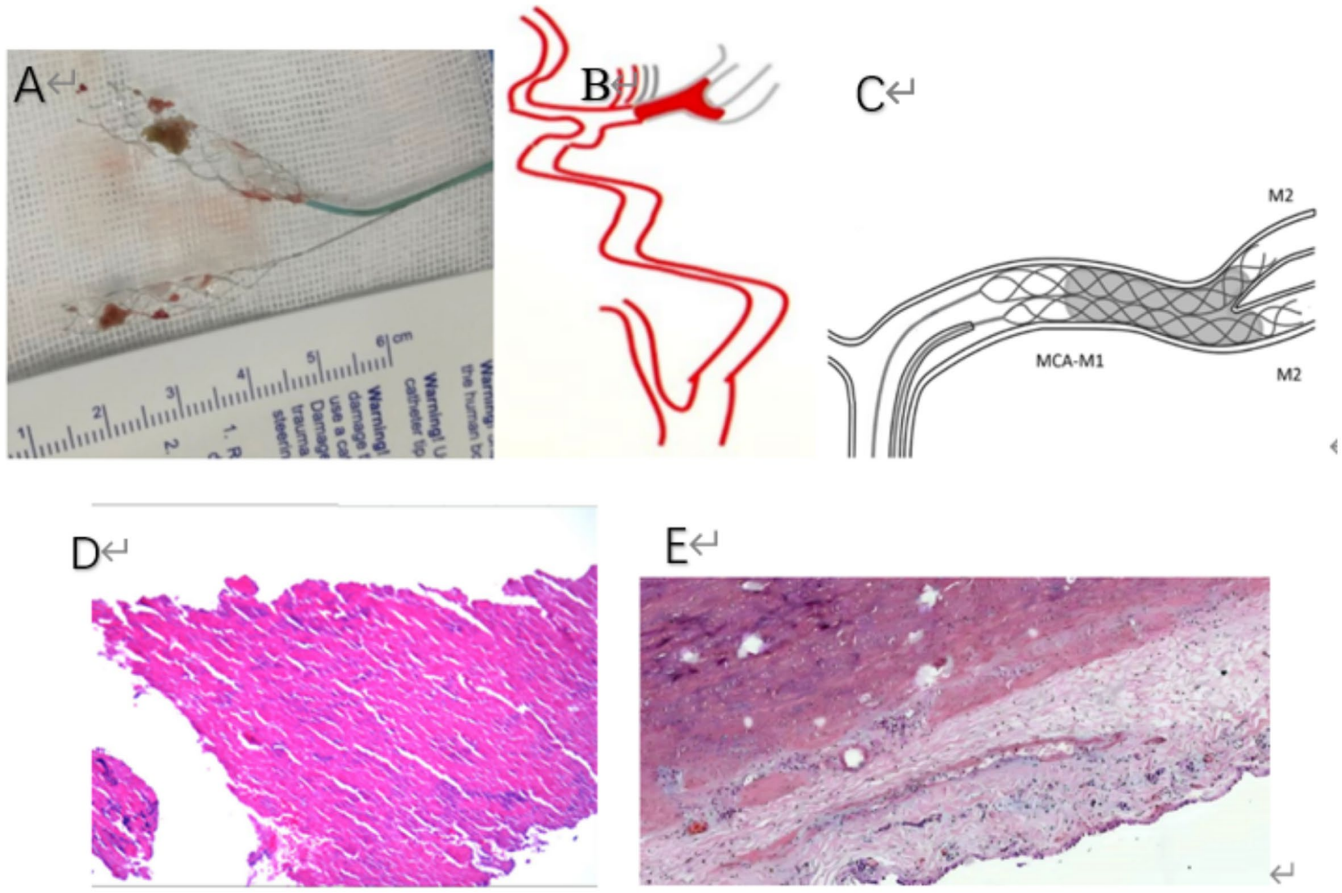
**(A)** The clot was successfully extracted by using a dual-stent technique. **(B)** A schematic representation depicts a thrombus obstructing the middle cerebral artery and extending to its bifurcation site. **(C)** A schematic illustration demonstrates the concurrent deployment of dual-stent retrievers within a clot lodged in the M1 segment of the middle cerebral artery (MCA-M1). The distal ends of these retrievers are shown reaching into both branches of the MCA bifurcation, corresponding to the M2 segments. **(D)** The pathological examination following stent thrombectomy revealed a mass of gray-white tissue that was visually observed to have a fleshy and solid texture. The dimensions of the tissue were measured to be 0.4 cm in length, 0.3 cm in width, and 0.3 cm in height. Upon microscopic analysis, it was determined that the specimen consists predominantly of red blood cells, accounting for about 70% of the sample. The remaining 30% is made up of white blood cells, fibrin, and platelets. The examination was conducted at a magnification of 10 times using hematoxylin and eosin (HE) staining. **(E)** Pathological results after heart valve resection surgery, under the microscope, there was proliferation of fibrous tissue in the valve leaflets, hyaline degeneration, calcium salt deposition, small vessel proliferation, lymphocyte infiltration, and local thrombosis. Pathological diagnosis: (Aortic Valve) Chronic valvulitis. (HE × 10).

Cerebral embolic events (CEEs) are common complications of IE. Embolism of the middle cerebral artery is the most prevalent type of CEE, presenting with large emboli in a significant proportion of cases. MT is the standard care for acute ischemic stroke (AIS) due to large vessel occlusion (LVO); however, MT fails to achieve adequate recanalization in nearly one-third of these cases. Especially, LVO involving bifurcation is usually resistant and has a high clot burden, reducing the possibility of successful recanalization (Figure 1B). Rescue therapy using dual-stentrievers yields good results for clots refractory to single-stentriever treatment (Figure 1C).

A 56-year-old female patient presented with symptoms of right-sided limb weakness and dysarthria. She was admitted to the hospital 5 h after the onset of symptoms. A brain computed tomography (CT) scan was performed, which ruled out the presence of intracerebral hemorrhage. The patient reported intermittent low-grade fevers 2 months before hospitalization, peaking at a body temperature of 37.7°C. Her medical history was unremarkable for other conditions, such as hypertension, diabetes mellitus, coronary artery disease, or arrhythmia.

On admission, she was conscious and her vital signs were as follows: blood pressure 159/92 mmHg; pulse rate 76 beats/min; temperature 37.2°C, and respiration rate 19 per minute. Neurological objective assessment on admission showed that the patient was conscious, her head and eyeballs were deviated to the left, with mixed aphasia, right hemiplegia, and shallow nasolabial fold on the right side. She exhibited uncooperative tongue extension on examination. Her Right Babinski sign and Chaddock’s sign were positive. A grade 3/6 systolic ejection murmur was heard in the auscultation area of the aortic valve.

**Figure 2:**
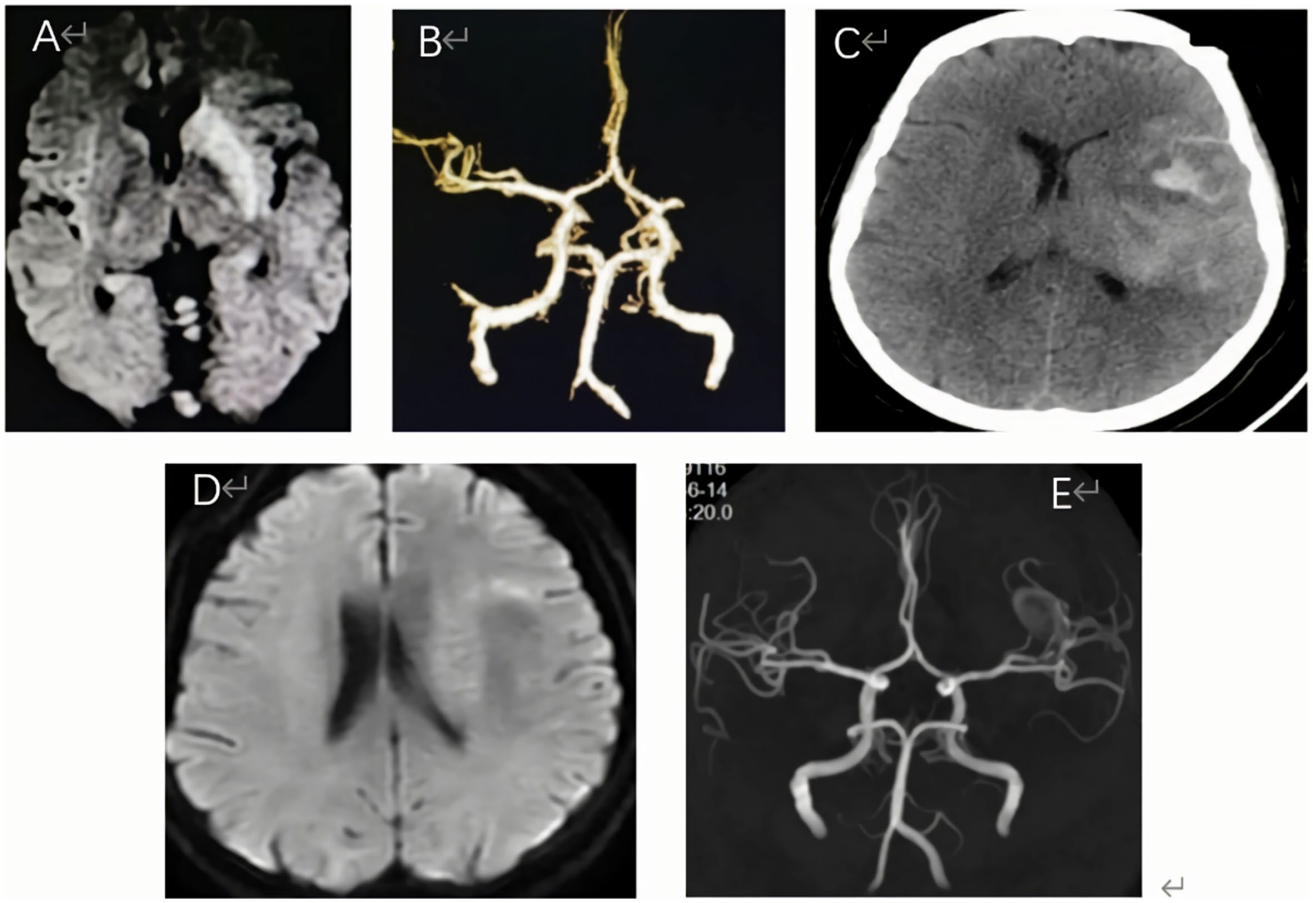
**(A)** Diffusion-weighted imaging (DWI) demonstrated prominent hyperintense signals within the basal ganglia and caudate nucleus, indicating potential acute ischemic changes. **(B)** Magnetic resonance angiography (MRA) confirmed the presence of an occlusion in the left middle cerebral artery. **(C)** A non-contrast CT head scan found contrast extravasation, and no intracranial hemorrhage or hyperperfusion. **(D)** Three days after oneset, brain MRI showed significant improvement in the high signal on the basal ganglia, but no significant increase of infarcted tissue. **(E)** Brain MRA indicated complete recanalization of the left MCA on the third day after operation.

She was admitted with an NIHSS score of 16, and the electrocardiogram demonstrated normal sinus rhythm and normal axis without any ischemic changes or pathological Q waves. Routine blood tests, bleeding and clotting times, blood glucose levels, and kidney function were all within normal limits. Magnetic resonance imaging (MRI) of the brain revealed hyperintense signals on T1-weighted and T2-weighted images in the left basal ganglia and caudate nucleus (Figure 2A). Magnetic resonance angiography (MRA) confirmed an occlusion in the left middle cerebral artery (Figure 2B). Further evaluation indicated a distal thrombus in the left middle cerebral artery (MCA), which extended to both the superior and inferior divisions.

Consequently, the diagnosis of acute cerebral infarction was confirmed. Additionally, the patient presented with an unexplained fever.

**Figure 3:**
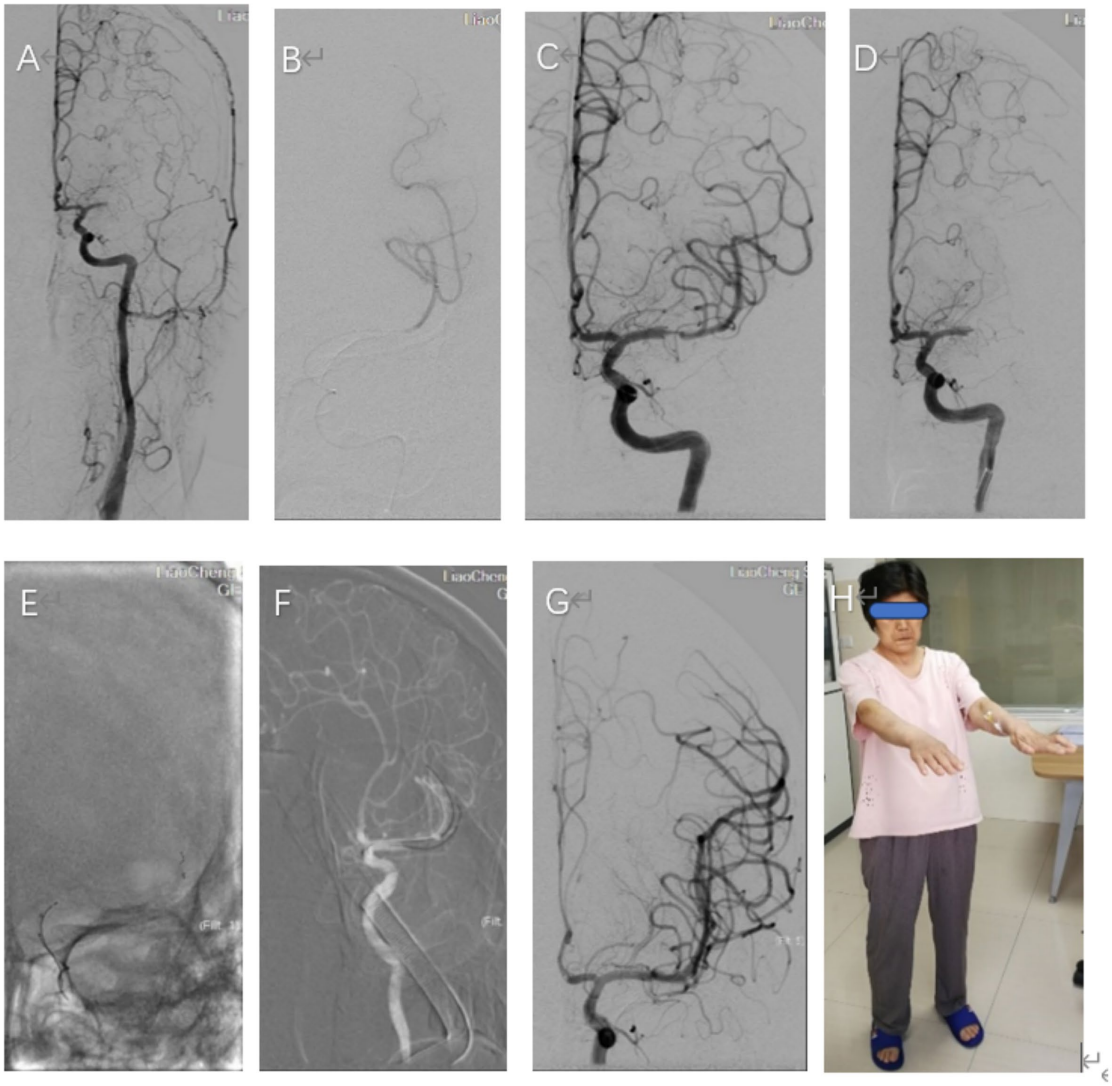
**(A)** A frontal angiogram showing, Left carotid angiogram confirmed occlusion of the left middle cerebral artery (MCA, M1 segment). **(B)** The presence of the distal true lumen was verified through the administration of contrast media via a microcatheter, successfully visualizing the lumen’s continuity. **(C)** Upon the full deployment of the stent, a partial restoration of blood flow was achieved in the left middle cerebral artery. The stent used was a 4 mm × 20 mm SOLITAIRE-FR stent. **(D)** Persistent occlusion of the left MCA after three passes with the single-stent retriever, and observed in the angiographic images. **(E,F)** The deployment of two stents within the M2 trunk’s superior and inferior branches was visualized through a fluoroscopic anteroposterior projection, which revealed the positioning of the stent retrievers in the left middle cerebral artery (MCA). Post-deployment, a contrast agent was injected to assess the stentriever positioning and vascular patency. **(G)** A frontal angiographic image demonstrates the full restoration of blood flow in the left middle cerebral artery (MCA), resulting in a modified Thrombolysis in Cerebral Infarction (mTICI) score of 3, indicating optimal reperfusion. **(H)** At the time of discharge, the patient’s neurological status was assessed using the National Institutes of Health Stroke Scale (NIHSS), which resulted in a score of 2, indicating mild stroke severity. Additionally, her functional outcome was evaluated with the Modified Rankin Scale (MRS), yielding a score of 2, suggesting slight disability affecting daily activities.

Beyond the time window of intravenous thrombolysis upon her admission, the patient was transferred to the angiosuite. With the consent of the patient’s family, an emergency cerebral angiography was performed, followed by endovascular thrombectomy. The occlusion of the left middle cerebral artery was identified (Figure 3A). Following an unsuccessful attempt with a single-stent thrombectomy, a dual-stent thrombectomy strategy was adopted, and the blood clot was successfully removed (Figure 1A). This intervention led to successful recanalization of the affected artery, achieving a modified thrombolysis in cerebral infarction (mTICI) score of 3 (Figure 3G).

After completing the active therapeutic intervention, the patient exhibited a noticeable improvement in her condition. Physical examination revealed a minor reduction in the fluency of her speech, while the muscle strength of her right limbs was rated at grade 5 on the medical scale. The patient was deemed fit for discharge after recovery (Figure 3H). Upon discharge, her National Institutes of Health Stroke Scale (NIHSS) score and her Modified Rankin Scale (MRS) score were both 2 (Figure 3H).

Endovascular treatment was as follows: All procedures were conducted by an experienced neurointerventionist using a monoplane angiography system. A 6F guiding support catheter (ev3, Plymouth, MN) was introduced into the Petrous segment of the left internal carotid artery. Then, a Rebar 18 microcatheter (ev3, Plymouth, MN) was navigated over the 300 cm long 0.014-in ASAHI guidewire (Asahi Intecc Medical, Japan), beyond the distal end of the occlusive clot under roadmap guidance. After withdrawing the micro-guidewire, the length of the occlusion and the distal lumen of the lesion was confirmed through microcatheter angiography (Figure 3B). After the complete release of the stent (Solitaire-FR, 4 mm × 20 mm, Medtronic), we observed the partial recanalization of the left middle cerebral artery (Figure 3C). Thrombectomy with three passes of the Solitaire-FR device 4 mm × 20 mm in the inferior MCA trunk was unsuccessful, and a left MCA bifurcation clot was observed (Figure 3D). At this point, we decided to employ a novel strategy for MT incorporating two Solitaire FR devices. A 6F guiding catheter could not be placed at the same time at two Rebar-18 microcatheters. Therefore, the Rebar-18 microcatheter was first placed in the M2 segment of the left MCA through the superior MCA trunk, while a Solitaire-FR 4 mm × 20 mm was placed such that the proximal end of the stent did not cover the bifurcation. Subsequently, the microcatheter was completely removed from the 6F guiding, leaving a bare Solitaire-FR inside the 6F guiding. Then, the inferior MCA trunk was catheterized with the same microcatheter and the Solitaire-AB 6 mm × 30 mm was unfolded by withdrawing the microcatheter (Figures 3E,F). When the microcatheter tip was nearly aligned with the bifurcation, we pulled the first Solitaire-FR to engage the clot. We continued to withdraw the microcatheter to position the second Solitaire FR in parallel (Figure 1C). Then, both stents were slowly pulled together into the 6F guiding under continuous aspiration. As resistance was felt while retracting the stent retriever, the entire assembly was slowly withdrawn under continuous aspiration. Subsequent follow-up angiograms showed the mTICI 3 reperfusion of MCA (Figure 3G). The interval between groin puncture and final revascularization was 98 min. After endovascular treatment, a CT scan confirmed no intracranial hemorrhage or hyperperfusion (Figure 2C). Brain MRI showed significant improvement in the high T1/T2 signal on the left basal ganglia (Figure 2D). MRA indicated complete recanalization of the left MCA on the third postoperative day (Figure 2E).

Further diagnostic assessments were conducted during the hospital stay. The blood culture test yielded a positive result for group streptococcus. Additionally, the level of antistreptolysin O was notably increased, reaching a titer of 263.72 IU/mL. In histopathological examinations, microscopic examination revealed that red blood cells constituted approximately 70%, while white blood cells, fibrin, and platelets collectively comprised 30%. The results were consistent with the study conducted by Thiene G et al. (1). A transesophageal echocardiogram (TEE) indicated the presence of thickened aortic valve leaflets accompanied by unusual growth, suggesting a high likelihood of vegetation. Consistently, we observed aortic regurgitation. Notably, a 9 by 10-mm mass was detected at the aortic valve’s base (See Supplementary Video). The patient continued to experience intermittent, mild fevers, with temperatures fluctuating between 37.5 and 37.9°C.

The patient was diagnosed with a cerebral embolism, which was attributed to subacute bacterial endocarditis. Consequently, the patient underwent aortic valve replacement. We scheduled a heart valve replacement surgery. The pathological results can be seen in Figure 1E. Postoperatively, the patient was consistently administered warfarin and underwent regular follow-up assessments for 2 years. To date, the patient’s health status has been favorable.

## Discussion

The incidence rate of IE embolism is 22–50%. It can affect all arteries, with cerebral artery embolism being the most common type (2). This type presents mostly with large embolus, manifesting as single or multiple lesions on imaging. The prognosis of most patients remains poor. MT has shown a significant benefit for patients with AIS of large intracerebral vessels (3). Rescue therapy using two simultaneous stentrievers yields good results for patients with clots refractory to MT with a single stentriever (4–6); During the actual operation, left internal carotid arteriography in the frontal projection showed left middle cerebral artery occlusion in this study, extending to both divisions. After the failure of single-stent thrombectomy, we combined the strategy adopted by Li et al. (5) and Cabral et al. (6) and used dual-stentriever thrombectomy as a rescue therapy for bifurcation occlusion. Consequently, we selected an alternative approach, employing a dual-stent technique to accomplish thrombectomy. Simultaneously, the excised vegetation was immediately removed and subjected to histopathological studies (see Figure 1A).

The presence of a high-density sign in a portion of the left cerebral hemisphere on postoperative CT images was attributed to the extravasation of the contrast agent. No severe complications were observed after the procedure. This patient’s TEE showed that the vegetation size was 9 mm × 10 mm. Based on the results of Mohananey et al. (7) and Papadimitriou et al. (8), there is an increased risk of re-embolization and mortality during hospitalization if the size exceeds 10 mm. We scheduled the patient for elective open-chest heart valve replacement surgery. The pathological results can be seen in Figure 1E. Under the microscope, we observed the proliferation of fibrous tissue in the valve leaflets, hyaline degeneration, calcium salt deposition, small vessel proliferation, lymphocyte infiltration, and local thrombosis. The pathological diagnosis was chronic valvulitis of the aortic valve (Figure 1D, HE×10).

Positive blood culture results of IE patients are risk factors for cerebrovascular diseases (9). Early valve replacement significantly reduces the risk of recurrence and death. The results of this case report showed Streptococcus spp. as the causative microbe, consistent with the findings of Misfeld et al. (10) Therefore, we performed early valve replacement surgery to prevent related adverse complications.

Therefore, as a rescue technique to MT technique, dual-stent thrombectomy has a high recanalization rate in selected cases and has many advantages. First, it acts as a temporary bypass by allowing the immediate restoration of flow through the clot by expanding the stent within the clot (11). Second, the dual-stent increases the degree of stent expansion, which may reflect the ability of the dual-stent thrombectomy technique to facilitate the device-clot interaction (12). Third, the dual-stent thrombectomy technique results in a longer device surface, which can enhance the device purchase distal to the clot, increasing the chances of removing the clot (13) (Figure 1C). Vega P et al. (14) found that dual-stentriever thrombectomy yields high rates of successful recanalization after the first pass with a low rate of complications, suggesting that it can be an effective and safe first-line treatment for M1 and TICA occlusions. Nevertheless, larger prospective studies are needed to validate the feasibility and safety of this strategy and determine its ability to improve clinical outcomes.

Our search did not find any randomized trials investigating the efficacy and safety of thrombectomy in IE associated with AIS. Moreover, there are few reported cases of thrombolysis or MT in this setting. Therefore, we can only draw conclusions based on some retrospective studies. Our conclusions may not be comprehensive, but they are reasonable inferences based on existing data and experience. This case study demonstrated that the dual-stent MT could be a viable and relatively safe strategy for LVO strokes subsequent to IE. These findings underscore the importance of prompt diagnosis, proactive intervention, and the necessity of an experienced multidisciplinary team to ensure optimal management and outcomes.

## Data Availability

The original contributions presented in the study are included in the article/Supplementary material, further inquiries can be directed to the corresponding author.

## References

[1] Thiene G Basso C. Pathology and pathogenesis of infective endocarditis in native heart valves. Cardiovasc Pathol. (2006) 15:256–63. doi: 10.1016/j.carpath.2006.05.009 16979032

[2] Ong E Mechtouff L Bernard E Cho TH Diallo LL Nighoghossian N. Thrombolysis for stroke caused by infective endocarditis: an illustrative case and review of the literature. J Neurol. (2013) 260:1339–42. doi: 10.1007/s00415-012-6802-1, PMID: 23292203

[3] Goyal M Menon BK van Zwam WH Dippel DW Mitchell PJ Demchuk AM. HERMES collaborators. Endovascular thrombectomy after large-vessel ischaemic stroke: a meta-analysis of individual patient data from five randomised trials. Lancet. (2016) 387:1723–31. doi: 10.1016/S0140-6736(16)00163-X, PMID: 26898852

[4] Aydin K Barburoglu M Oztop Cakmak O Yesilot N Vanli ENY Akpek S. Crossing Y-Solitaire thrombectomy as a rescue treatment for refractory acute occlusions of the middle cerebral artery. J Neurointerv Surg. (2019) 11:246–50. doi: 10.1136/neurintsurg-2018-014288, PMID: 30194110

[5] Li Z Liu P Zhang L Zhang Y Fang Y Xing P. Y-stent rescue technique for failed thrombectomy in patients with large vessel occlusion: a case series and pooled analysis. Front Neurol. (2020) 11:924. doi: 10.3389/fneur.2020.00924, PMID: 32973671 PMC7481477

[6] Cabral LS Mont'Alverne F Silva HC Passos Filho PE Magalhães PSC Bianchin MM. Device size selection can enhance Y-stentrieving efficacy and safety as a rescue strategy in stroke thrombectomy. J Neurointerv Surg. (2022) 14:558–63. doi: 10.1136/neurintsurg-2021-017751, PMID: 34233944

[7] Mohananey D Mohadjer A Pettersson G Navia J Gordon S Shrestha N. Association of vegetation size with embolic risk in patients with infective endocarditis: a systematic review and meta-analysis. JAMA Intern Med. (2018) 178:502–10. doi: 10.1001/jamainternmed.2017.8653, PMID: 29459947 PMC5876809

[8] Papadimitriou-Olivgeris M Guery B Ianculescu N Dunet V Messaoudi Y Pistocchi S. Role of cerebral imaging on diagnosis and management in patients with Suspected Infective Endocarditis. Clin Infect Dis. (2023) 77:371–9. doi: 10.1093/cid/ciad192, PMID: 36999313 PMC10425197

[9] Ying M Hong Z Yanyan L Shinong P Qingping M. Clinical and imaging characteristics of infectious endocarditis complicated with cerebrovascular disease. Chin J Mod Med. (2008) 18:5. doi: 10.3969/j.issn.1005-8982.2008.06.024

[10] Misfeld M Girrbach F Etz CD Binner C Aspern KV Dohmen PM. Surgery for infective endocarditis complicated by cerebral embolism: a consecutive series of 375 patients. J Thorac Cardiovasc Surg. (2014) 147:1837–46. doi: 10.1016/j.jtcvs.2013.10.076, PMID: 24837722

[11] Patro SN Iancu D. Dual-stent retrieval for mechanical thrombectomy of refractory clot in acute stroke as a rescue technique. CMAJ. (2017) 189:E634–7. doi: 10.1503/cmaj.160472 28461375 PMC5415391

[12] Imahori T Miura S Sugihara M Mizobe T Aihara H Kohmura E. Double stent retriever (SR) technique: a novel mechanical Thrombectomy technique to facilitate the device-clot interaction for refractory acute cerebral large vessel occlusions. World Neurosurg. (2020) 141:175–83. doi: 10.1016/j.wneu.2020.05.268 32522654

[13] Haussen DC Al-Bayati AR Grossberg JA Bouslama M Barreira C Bianchi N. Longer stent retrievers enhance thrombectomy performance in acute stroke. J Neurointerv Surg. (2019) 11:6–8. doi: 10.1136/neurintsurg-2018-013918, PMID: 29858398

[14] Vega P Murias E Jimenez JM Chaviano J Rodriguez J Calleja S. First-line double Stentriever Thrombectomy for M1/TICA occlusions: initial experiences. Clin Neuroradiol. (2022) 32:971–7. doi: 10.1007/s00062-022-01161-2, PMID: 35416489 PMC9744691

